# First Impressions Matter: Improving Resident Doctor Induction at Stobhill Mental Health Campus

**DOI:** 10.1192/bjo.2025.10432

**Published:** 2025-06-20

**Authors:** Alys Wyn Roberts, Lindsey McKeown, Martha Kelly

**Affiliations:** NHS Glasgow and Clyde, Glasgow, United Kingdom

## Abstract

**Aims:** The Scottish training survey 2024 and General Medical Council national training survey 2024 showed that the general psychiatry training post at the Stobhill Mental Health Campus (SMHC) scored lower in the induction domain compared with the national average. The aim of this quality improvement project was to identify areas of induction that required improvement, implement this improvement, and deliver a new upgraded induction.

**Methods:** An eleven-question survey was devised which included nine 5-point Likert scale questions and two free text questions. In November 2024 the survey was sent to current resident doctors to gather feedback about the original induction. There were eight respondents, and based on this feedback a new induction was set up. The existing presentation was replaced with seminar-style discussion and a duty doctor simulation session, led by current psychiatry trainees. This focused on site-specific scenarios designed to familiarize new trainees with common challenges.

In December 2024 five new resident doctors received this new trainee-led induction and following this they completed the survey. Again, based on this feedback the induction process was adjusted and in February 2025 five new resident doctors completed the updated induction and provided feedback via the survey.

**Results:** The feedback from doctors who had received the original induction was poor, and there was marked improvement in responses for both the December 2024 and February 2025 induction.

Of those who received the original induction, only 14% agreed that the induction had prepared them well for their first on-call shift at SMHC. This improved to 100% and 80% with the implementation of the new induction. There were also marked improvements in the number of respondents that agreed that induction helped them understand the post’s roles and responsibilities, as well as their understanding of the electronic handover document. Improvements were also evident in the resident doctors feeling more confident in their ability to contact senior psychiatry colleagues and other acute specialities.

**Conclusion:** The new induction format has significantly improved the induction experience at SMHC, and resident doctors now feel more prepared for on-call shifts. We hope that this will eventually improve trainee morale, overall satisfaction with the training post and also improve clinical care.

This new version of induction will continue to be delivered, and feedback will be collected to ensure ongoing improvement. We await the results of the Scottish training survey and GMC national training survey to see whether our data is reflected in their results.

